# Evolution of Opsin Genes in Caddisflies (Insecta: Trichoptera)

**DOI:** 10.1093/gbe/evae185

**Published:** 2024-08-23

**Authors:** Ashlyn Powell, Jacqueline Heckenhauer, Steffen U Pauls, Blanca Ríos-Touma, Ryoichi B Kuranishi, Ralph W Holzenthal, Ernesto Razuri-Gonzales, Seth Bybee, Paul B Frandsen

**Affiliations:** Department of Plant and Wildlife Sciences, Brigham Young University, Provo, UT, USA; LOEWE Centre for Translational Biodiversity Genomics, Frankfurt, Germany; Senckenberg Research Institute and Natural History Museum Frankfurt, Frankfurt, Germany; LOEWE Centre for Translational Biodiversity Genomics, Frankfurt, Germany; Senckenberg Research Institute and Natural History Museum Frankfurt, Frankfurt, Germany; Facultad de Ingenierías y Ciencias Aplicadas, Ingeniería Ambiental, Grupo de Investigación en Biodiversidad, Medio Ambiente y Salud, Universidad de Las Américas, Quito, Ecuador; Graduate School of Science, Chiba University, Chiba, Japan; Kanagawa Institute of Technology, Kanagawa, Japan; Department of Entomology, University of Minnesota, St Paul, MN, USA; Senckenberg Research Institute and Natural History Museum Frankfurt, Frankfurt, Germany; Department of Biology, Brigham Young University, Provo, UT, USA; Department of Plant and Wildlife Sciences, Brigham Young University, Provo, UT, USA

**Keywords:** caddisflies, evolution, opsins, visual systems, Trichoptera, insects

## Abstract

Insects have evolved complex and diverse visual systems in which light-sensing protein molecules called “opsins” couple with a chromophore to form photopigments. Insect photopigments group into three major gene families based on wavelength sensitivity: long wavelength (LW), short wavelength (SW), and ultraviolet wavelength (UV). In this study, we identified 123 opsin sequences from whole-genome assemblies across 25 caddisfly species (Insecta: Trichoptera). We discovered the LW opsins have the most diversity across species and form two separate clades in the opsin gene tree. Conversely, we observed a loss of the SW opsin in half of the trichopteran species in this study, which might be associated with the fact that caddisflies are active during low-light conditions. Lastly, we found a single copy of the UV opsin in all the species in this study, with one exception: *Athripsodes cinereus* has two copies of the UV opsin and resides within a clade of caddisflies with colorful wing patterns.

**Significance**

While opsin evolution in some insect groups has been well-characterized, it has never been studied across caddisflies. Our findings provide insight into the diversity of opsins in caddisflies and form a basis for further research into the evolutionary drivers and complex visual systems in Trichoptera.

## Introduction

Within the visual system, the ability to perceive light is critical and plays an essential role in the life histories of insects, including finding food, avoiding predators, and selecting a mate (van der Kooi et al. 2021). Light perception occurs primarily within three different types of visual organs in insects: the stemmata of larvae and the ocelli and compound eyes of adults (van der Kooi et al. 2021; Guignard et al. 2022). Upon light absorption, photoreceptors within the eyes—which contain opsin proteins and chromophores—change their configuration from a resting state to a signaling state, thereby indicating a physiological response (Shichida and Matsuyama 2009). Insect visual opsins form three major gene clades based on their peak wavelength sensitivity, namely, long wavelength (LW; 500 to 600 nm), short wavelength (SW; 400 to 500 nm), and ultraviolet wavelength (UV; 300 to 400 nm; Feuda et al. 2016; Lord et al. 2016; van der Kooi et al. 2021). Many insect groups possess an additional opsin type, Rhodopsin 7 (RH7), which does not have a known function in most insect groups but was found to be involved in circadian rhythms in *Drosophila* (Senthilan and Helfrich-Förster 2016; Ni et al. 2017).

Insects typically possess one or more copies of each opsin type. Moreover, multiple cases of gene duplications and losses have been observed throughout insect opsin evolution (Spaethe and Briscoe 2004; Frentiu et al. 2007; Sison-Mangus et al. 2008; Briscoe et al. 2010; French et al. 2015; Futahashi et al. 2015; Feuda et al. 2016; Lord et al. 2016; Sharkey et al. 2017; Suvorov et al. 2017; Sondhi et al. 2021; Guignard et al. 2022; Friedrich 2023; Mulhair et al. 2023). These duplications, and subsequent diversification, of visual opsin genes are the primary mechanisms of evolution that lead to greater visual capacity and flexibility (Frentiu et al. 2007; Suvorov et al. 2017; Friedrich 2023) and are usually linked to a particular life-history strategy, living environment, or light condition (French et al. 2015; Futahashi et al. 2015; Lord et al. 2016; Sharkey et al. 2017, 2023; Sondhi et al. 2021; Guignard et al. 2022). While the evolution of opsins has been relatively well studied in some insect orders, opsin genes in caddisflies (Insecta: Trichoptera) have only been characterized in a single species as part of a broader comparative study across insects (Guignard et al. 2022).

**Fig. 1.**
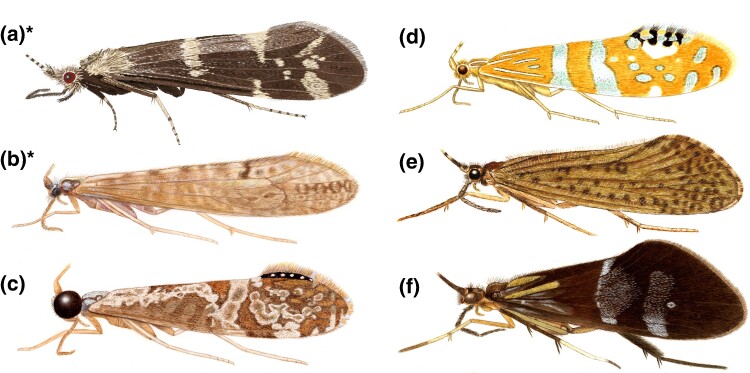
Adult caddisfly illustrations showing varying eye sizes and diverse wing colorations and patterns. a) *Athripsodes cinereus* (Leptoceridae); b) *N. paramo* (Leptoceridae); c) *Nectopsyche nigricapilla* (Leptoceridae); d) *Nectopsyche ortizi* (Leptoceridae); e) *Banyallarga vicaria* (Calamoceratidae); f) *Phylloicus abdominalis* (Calamoceratidae). *Species included in this study. Illustrations by Julie Martinez and Ralph Holzenthal.

As eggs, larvae, and pupae, caddisflies mainly inhabit the benthic zone of freshwater habitats, but as adults, they occupy terrestrial environments adjacent to freshwater (Morse et al. 2019). There are two monophyletic suborders within Trichoptera characterized by differences in habitat, morphology, and silk use: Annulipalpia (retreat making) and Integripalpia (cocoon and case making; Frandsen et al. 2024). Adult caddisflies resemble small moths; yet, while most species have wings and bodies covered in small hairs instead of scales, a few species have brightly colored wings with red, orange, green, or silver regions (Fig. 1) due to the development of hairs into scales (Holzenthal et al. 2007). Additionally, adult caddisflies possess varying eye sizes, some much larger than others (Fig. 1). Presumably, such varied environments—both aquatic and terrestrial—and wing colorations require a plastic and diverse visual system. To gain an understanding of opsin evolution in caddisflies, we analyzed the occurrence and phylogenetic relationships of opsin genes from whole-genome assemblies across 25 caddisfly species (Table 1), representing the major evolutionary lineages within the order.

**Table 1:** Locations and quality scores of genome assemblies for each species

Species	Accession number	Publication	Contig N50 (bp)	Compleasm (recovered BUSCOs)
*Leptonema lineaticorne*	GCA_024500535.1	Heckenhauer et al. (2023)	14,931,587	C: 98.30% [S: 97.83%, D: 0.47%], F: 0.66%, I: 0.00%, M: 1.04%
*Hydropsyche tenuis*	GCA_009617725.1	Heckenhauer et al. (2019)	2,190,134	C: 97.93% [S: 97.65%, D: 0.28%], F: 0.89%, I: 0.00%, M: 1.18%
*Parapsyche elsis*	GCA_022651745.1	Frandsen et al. (2019), Heckenhauer et al. (2022)	5,591,679	C: 96.99% [S: 96.80%, D: 0.19%], F: 0.80%, I: 0.00%, M: 2.21%
*Arctopsyche grandis*	GCA_029955255.1	Frandsen et al. (2023)	6,470,670	C: 98.36% [S: 95.39%, D: 2.97%], F: 0.71%, I: 0.00%, M: 0.94%
*Plectrocnemia conspersa*	JAUTWC000000000	New	32,103,979	C: 97.74% [S: 97.32%, D: 0.42%], F: 0.61%, I: 0.00%, M: 1.65%
*Philopotamus ludificatus*	GCA_022495035.1	Heckenhauer et al. (2022)	35,449	C: 94.63% [S: 93.31%, D: 1.32%], F: 3.15%, I: 0.05%, M: 2.17%
*Stenopsyche tienmushanensis*	GCA_008973525.1	Luo et al. (2018)	1,296,863	C: 97.50% [S: 95.15%, D: 2.35%], F: 1.04%, I: 0.00%, M: 1.46%
*Agraylea sexmaculata*	GCA_022606485.1	Heckenhauer et al. (2022)	86,524	C: 95.67% [S: 90.96%, D: 4.71%], F: 1.04%, I: 0.00%, M: 3.30%
*Glossosoma conforme*	GCA_022606575.1	Heckenhauer et al. (2022)	2,212,131	C: 93.13% [S: 92.47%,D: 0.66%], F: 0.66%, I: 0.00%, M: 6.21%
*Atopsyche davidsoni*	GCA_022113835.1	Ríos-Touma et al. (2021)	14,095,054	C: 98.68% [S: 98.35%, D: 0.33%], F: 0.61%, I: 0.00%, M: 0.71%
*Atopsyche callosa*	Available on FigShare	New	25,586,909	C: 98.54% [S: 96.70%, D: 1.84%], F: 0.66%, I: 0.00%, M: 0.80%
*Himalopsyche phryganea*	GCA_022494535.1	Heckenhauer et al. (2019)	4,634,010	C: 98.21% [S: 97.83%,D: 0.38%], F: 0.71%, I: 0.00%, M: 1.08%
*Himalopsyche tibetana*	GCA_030503985.1	Heckenhauer et al. (2022)	28,889,006	C: 98.91% [S: 98.35%, D: 0.56%], F: 0.56%, I: 0.00%, M: 0.52%
*Rhyacophila brunnea*	Available on FigShare	New	1,306,779	C: 97.74% [S: 94.35%, D: 3.39%], F: 1.22%, I: 0.00%, M: 1.04%
*Micrasema minimum*	GCA_022494985.1	Heckenhauer et al. (2019)	69,526	C: 66.34% [S: 66.20%, D: 0.14%], F: 7.25%, I: 0.00%, M: 26.41%
*Lepidostoma basale*	GCA_022606425.2	Heckenhauer et al. (2019)	1,001,566	C: 98.16% [S: 97.55%, D: 0.61%], F: 0.85%, I: 0.05%, M: 0.94%
*Drusus annulatus*	GCA_022651775.1	Heckenhauer et al. (2019)	1,032,046	C: 97.92% [S: 97.50%, D: 0.42%], F: 1.08%, I: 0.00%, M: 0.99%
*Halesus radiatus*	GCA_022606495.2	Heckenhauer et al. (2019)	124,280	C: 91.20% [S: 89.69%, D: 1.51%], F: 4.38%, I: 0.05%, M: 4.38%
*Hesperophylax magnus*	GCA_026573805.1	Frandsen et al. (2023)	11,205,906	C: 98.68% [S: 96.56%, D: 2.12%], F: 0.61%, I: 0.00%, M: 0.71%
*Agrypnia vestita*	GCA_016648135.1	Olsen et al. (2021)	111,757	C: 91.25% [S: 83.10%, D: 8.15%], F: 4.14%, I: 0.09%, M: 4.52%
*Eubasilissa regina*	GCA_022840565.1	Kawahara et al. (2022)	29,378,647	C: 98.73% [S: 96.28%, D: 2.45%], F: 0.80%, I: 0.00%, M: 0.47%
*Nectopsyche paramo*	JAWQED000000000	New	1,090,281	C: 97.31% [S: 95.24%, D: 2.07%], F: 0.80%, I: 0.00%, M: 1.88%
*Athripsodes cinereus*	GCA_947579605.1	Darwin Tree of Life Project	948,465	C: 97.22% [S: 96.61%, D: 0.61%], F: 1.08%, I: 0.00%, M: 1.69%
*Odontocerum albicorne*	GCA_949825065.1	Darwin Tree of Life Project	387,033	C: 97.22% [S: 96.75%, D: 0.47%], F: 1.18%, I: 0.00%, M: 1.60%
*Limnocentropus insolitus*	Available on FigShare	New	33,230,923	C: 98.87% [S: 98.26%, D: 0.61%], F: 0.56%, I: 0.00%, M: 0.56%

Compleasm was run with the Endopterygota OrthoDB v10 BUSCO gene set and the results categories are as follows: C, complete; S, single; D, duplicated; F, fragmented Subclass 1 (only a portion of the gene is present in the assembly); I, fragmented Subclass 2 (different sections of the gene align to different locations in the assembly); M, missing.

## Results

### Opsin Distribution and Gene Tree

The number of opsin paralogs found within each species ranged from three to as many as nine (Fig. 3). Opsin diversity within Integripalpia (Fig. 3, Clade 2) was more variable than that of Annulipalpia (Fig. 3, Clade 1). For example, the number of LW opsins among species of Integripalpia ranged from one to five paralogs. While we recovered all three visual opsins in four of the seven species within “basal Integripalpia” (Fig. 3, Clade 2a), the three remaining species from the family Rhyacophilidae were found to have lost the SW opsin. Conversely, in the tube-case-making Integripalpia (Fig. 3, Clade 2b), the SW opsin was lost in all except *Nectopsyche paramo* and *Athripsodes cinereus*, both of which are from the family Leptoceridae.

**Fig. 2.**
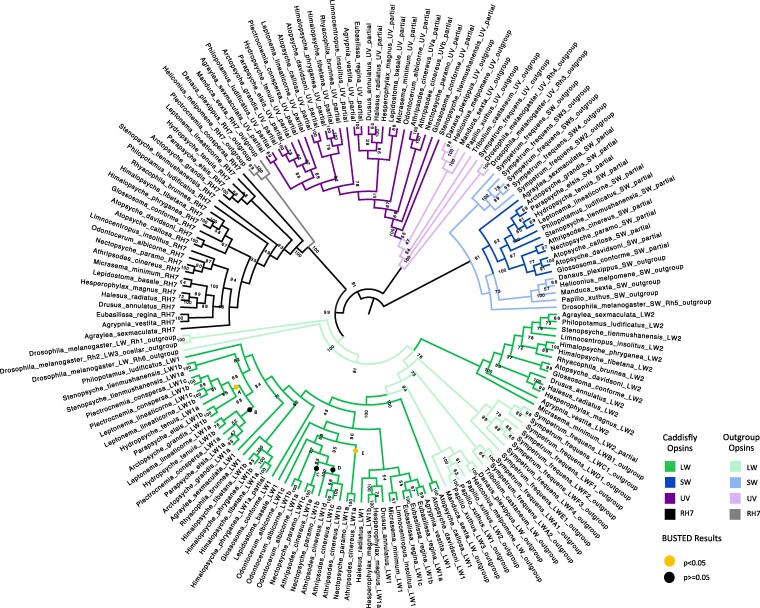
Opsin gene tree. Maximum likelihood tree from caddisfly and outgroup opsin DNA sequences. Nodes are labeled with bootstrap values and branches are colored by opsin type. The letters at the end of the node labels (e.g. LW1a, LW1b, etc.) denote multiple copies of that opsin type in a species. Clades marked with a dot (A to E) were tested for episodic diversifying selection with BUSTED (supplementary table S3, Supplementary Material online).

The opsin sequences formed four distinct clades in the gene tree corresponding to the LW, SW, UV, and RH7 opsin groups (Fig. 2). Within each opsin clade, the arrangements of suborders were maintained, but occasionally, interspecific relationships of opsin sequences did not match those in the established species tree (Frandsen et al. 2024). We found only minor differences between the coding sequence (CDS) and the peptide gene trees, primarily within the LW and UV opsin groups (supplementary fig. S2, Supplementary Material online). However, none of these areas of incongruence occurred across opsin classes and they were in areas of the trees with lower bootstrap support values (supplementary fig. S2, Supplementary Material online). The LW opsins formed two distinct clades in the opsin gene tree, a phenomenon observed in other insect orders (Futahashi et al. 2015; Feuda et al. 2016; Lord et al. 2016; Sharkey et al. 2017, 2023; Suvorov et al. 2017; Sondhi et al. 2021; Guignard et al. 2022, Mulhair et al. 2023). All species had at least 1 LW opsin in the LW1 clade, while only 14 species—distributed across both suborders—had an additional LW2 opsin (supplementary fig. S1, Supplementary Material online).

### Lineage-Specific Gene Duplication Events

We observed a few instances of paralogs that were paired in the opsin gene tree (Fig. 2) and adjacent to each other on the same contig in the genome assembly (supplementary table S1, Supplementary Material online), suggesting independent tandem duplication events. This was true for copies of the UV opsin in *A. cinereus* and the LW1 opsin in *Stenopsyche tienmushanensis*, *Leptonema lineaticorne*, *Plectrocnemia conspersa*, *Hesperophylax magnus*, *Eubasilissa regina*, *O. albicorne*, and *A. cinereus* (Fig. 2). Upon testing the branches of these duplicate opsins for selection with the Branch-Site Unrestricted Statistical Test for Episodic Diversification (BUSTED; Murrell et al. 2015; Kosakovsky Pond et al. 2020), we found evidence of episodic diversifying selection for three opsin paralogs: *L. lineaticorne* LW1c, *S. tienmushanensis* LW1b, and *E. regina* LW1b (supplementary table S3, Supplementary Material online).

We also found instances of gene duplication in the common ancestor of closely related species. For example, Clades A and B in Fig. 2 each have one or more LW1 opsin from the species *L. lineaticorne*, *Hydropsyche tenuis*, *Parapsyche elsis*, *Arctopsyche grandis*, and *P. conspersa*, all but the latter of which belong to the family Hydropsychidae. We tested for episodic diversifying selection using BUSTED (Murrell et al. 2015; Kosakovsky Pond et al. 2020) and found evidence of selection in the sequences in Clade A but not Clade B (Fig. 2, supplementary table S3, Supplementary Material online). Similarly, multiple duplication events occurred in the LW1 opsins in the lineage, leading to *N. paramo* and *A. cinereus*, both from the family Leptoceridae (Fig. 2, Clades C to E). Among these duplications, we found evidence of selection in the opsin sequences belonging to only Clade E (supplementary table S3, Supplementary Material online).

## Discussion

We searched across 25 caddisfly genome assemblies to determine the number and phylogenetic relationships of opsins in Trichoptera. Our results suggest that caddisfly opsin evolution is likely driven by life-history strategies and ambient light conditions as found in other insect orders (Briscoe et al. 2010; French et al. 2015; Futahashi et al. 2015, Feuda et al. 2016; Lord et al. 2016; Sharkey et al. 2017; Suvorov et al. 2017; Sondhi et al. 2021; Guignard et al. 2022; Mulhair et al. 2023).

We found some incongruencies between the gene tree and the species tree. Given the relatively small number of characters compared with the reference species tree, which was generated from genome-wide data (Frandsen et al. 2024), this is not unexpected and could be due to stochastic error from an undersampling of characters. This is also evidenced by lower bootstrap values in areas of the tree that were incongruent with the species tree (Fig. 2).

**Fig. 3.**
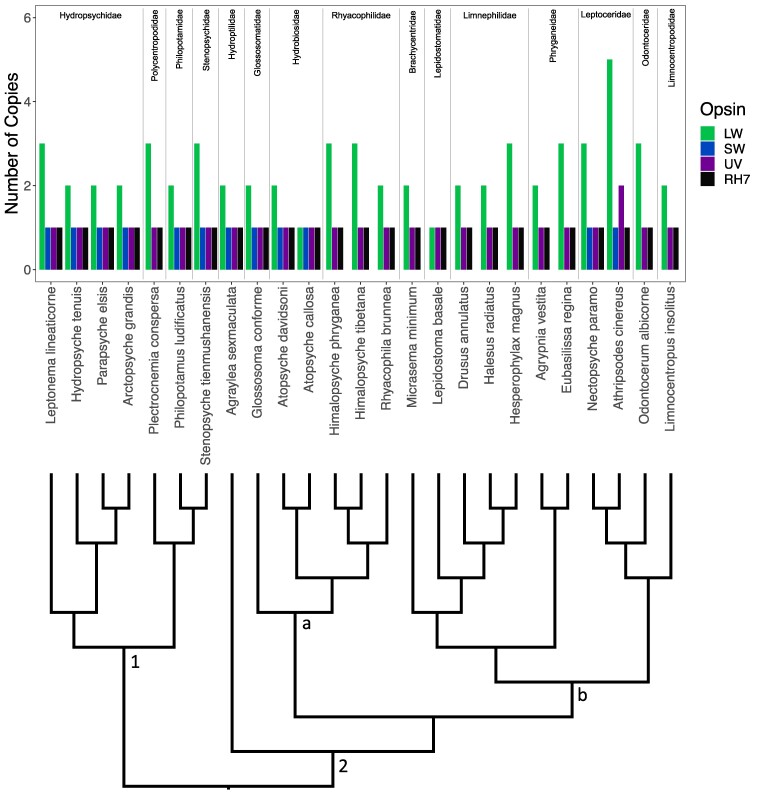
Opsin counts by species. A bar plot of the number of opsin copies in each caddisfly genome. The bars are colored by opsin type. The species are ordered by the species phylogeny indicated below the bar plot, which is based on a recent study that examined the caddisfly phylogeny in depth (Frandsen et al. 2024). Families are labeled above the bar plot. Suborders are labeled within the phylogeny as follows: (1) Annulipalpia—retreat makers; (2) Integripalpia—cocoon- and tube-case makers; (a) (and *Agraylea sexmaculata*) basal Integripalpia—cocoon makers; (b) tube-case-making Integripalpia.

The distribution of opsins within Annulipalpia, the fixed-retreat makers, was relatively invariable. Interestingly, their ecological distributions are also less varied; most species inhabit fast-moving streams as larvae and are short lived in the riparian zone as adults. While there were a few LW1 duplications in this suborder, the only loss we observed was the SW opsin gene in *P. conspersa* (Fig. 3). When searching for opsin genes, we found a sequence highly similar to the SW opsin in this species; however, we excluded it from the dataset due to the presence of stop codons. Given that the genome assembly of *P. conspersa* was of high quality, we hypothesize that this is likely a true loss (Table 1).

The SW opsin gene was also lost in Integripalpia within the basal cocoon-making family, Rhyacophilidae, and within the majority of the clade of tube-case makers (Fig. 3, Clade 2b). Loss of the SW opsin in other groups such as the American cockroach (French et al. 2015) and Neuropteroidea (Lord et al. 2016; Sharkey et al. 2017, 2023) is hypothesized to be associated with the low-light environments in which the ancestors of these insects lived. Caddisflies are primarily crepuscular and thus are most active during low-light conditions, which may be related to the loss of the SW opsin in Rhyacophilidae and in many tube-case-making caddisflies.

Interestingly, in contrast to most species within the tube-case makers, we were able to find the SW opsin in both *N. paramo* and *A. cinereus.* The latter was also the only species with two UV opsins and the highest number of LW opsins. Both *N. paramo* and *A. cinereus* belong to the family Leptoceridae, a family with known sexual eye dimorphism (Gullefors and Petersson 1993), long adult antennae, and many genera with intricate wing patterns and brightly colored or iridescent wing hairs and scales (Fig. 1; Holzenthal et al. 2007). The high number of opsins and the presence of the SW opsin could be related to the variety in wing coloration and patterns in some species of Leptoceridae. Future work should combine more sampling within this interesting area of the caddisfly phylogeny with gene expression and physiological data to better model the visual system of Trichoptera and to test hypotheses related to color vision and opsin diversity.

To further investigate the role of the duplication events that we observed in the LW and UV opsin groups, we tested paralogs in these areas of the tree for positive selection. We found evidence of episodic diversifying selection in some LW1 opsin paralogs but did not detect evidence of selection in the UV opsin paralogs (supplementary table S3, Supplementary Material online). In each instance when a paralog was found to be under selection, the duplicate paralog was not found to be under selection, possibly suggesting a route to neofunctionalization in those copies undergoing diversifying selection. Recent work in Lepidoptera and Hemiptera has identified instances of family- and species-specific duplications of visual opsins leading to adaptations that extend visual capacity (Spaethe and Briscoe 2004; Frentiu et al. 2007; Sison-Mangus et al. 2008; Briscoe et al. 2010; Feuda et al. 2016; Finkbeiner and Briscoe 2021; Friedrich 2023; Mulhair et al. 2023). Denser taxon sampling in future work can help clarify the evolutionary timing of duplication events and the mechanisms and role of selection that we uncovered.

Here, we conducted the first comprehensive study of visual opsins in Trichoptera. We found that the species with the highest diversity of opsins were derived from the group with known sexual eye dimorphism (Gullefors and Petersson 1993) and, which also contains species of the most colorful and intricately patterned wings, the Leptoceridae. Opsin evolution in caddisflies may also have been driven by life-history strategies and the low-light conditions during which caddisflies are active. The findings of this study provide a basis for future research on the diverse and complex visual systems in Trichoptera.

## Materials and Methods

We assessed 25 species of Trichoptera using five newly assembled genomes (details in supplemental note S1, Supplementary Material online) and 20 publicly available genome assemblies (Table 1). Using the 1,000 opsin sequences found by Guignard et al. (2022), we performed a tBLASTn search of opsin sequences against each caddisfly genome, keeping hits with an *e*-value <10^−40^. The resulting opsin sequences were extracted from their corresponding genomes using Pyfaidx (Shirley et al. 2015). We filtered redundant hits from multiple queries by extracting the widest window from each contig and then classifying the gene phylogenetically downstream.

We performed gene prediction using AUGUSTUS v3.4.0 (Stanke, et al. 2006), followed by a BLASTp search against the online NCBI databases, maintaining only hits similar to other insect opsins. We then manually checked the annotations in Geneious Prime v2023.0.4 (https://www.geneious.com), using outgroup sequences as a guide, to ensure that the entire gene was correctly annotated (see supplementary note S1, Supplementary Material online, for more details). We included opsin sequences from a variety of insect orders for outgroup comparison: Lepidoptera (*Danaus plexippus*, *Heliconius melpomene*, *Manduca sexta*, *Papilio xuthus*), Odonata (*Sympetrum frequens*), Diptera (*Drosophila melanogaster*), and Coleoptera (*Tribolium castaneum*), all accessed through GenBank (Benson et al. 2013). We performed additional searches for opsin genes to further verify the absence of the SW and LW2 opsins in many species (see supplementary note S1, Supplementary Material online, for more details). We also provide supplementary tables (supplementary table S2a to c, Supplementary Material online) with information on the completeness of each visual opsin sequence.

### Opsin Gene Tree Reconstruction

To determine phylogenetic relationships among opsin sequences, we first aligned the opsin peptide sequences using MAFFT v7.487 (Katoh and Standley 2013) and created a codon alignment with PAL2NAL v14.1 (Suyama et al. 2009). We performed phylogenetic reconstruction on both the CDS and the peptide alignments by first selecting the best substitution model using ModelFinder (Kalyaanamoorthy et al. 2017; Minh et al. 2020) and then performing a maximum likelihood tree search with 1,000 UltraFast bootstrap replicates corrected with the bootstrap nearest neighbor interchange option enabled to guard against the risk of overestimating bootstrap support (-bb 1000 -bnni). We viewed the resulting trees in FigTree v1.4.4 (Rambaut 2018). To highlight differences between the CDS and the peptide trees, we created a face-to-face comparison in R with ggtree v3.2.1 (Yu et al. 2016) and ggplot2 v3.3.5 (Wickham 2016; supplementary fig. S2, Supplementary Material online).

### Selection Analysis

To assess the duplications in the LW and UV opsin groups, we created separate codon alignments for both the LW and the UV opsin groups with MAFFT v7.487 (Katoh and Standley 2013) and PAL2NAL v14.1 (Suyama et al. 2009) and then tested for episodic diversifying selection using BUSTED as implemented in HyPhy (Murrell et al. 2015; Kosakovsky Pond et al. 2020). We tested branches of species-specific duplicated opsins individually as well as five deeper duplication events (Fig. 2, Clades A to E) and reported the resulting *P*-values in supplementary table S3, Supplementary Material online.

## Supplementary Material

evae185_Supplementary_Data

## Data Availability

Genome assemblies are available on GenBank at their respective accession numbers. Gene alignments for the opsins, gff files, tree files, and new genome assemblies are available on FigShare at the following DOI: https://doi.org/10.6084/m9.figshare.24164217.v1.
