# Supplementary figures and images for: Evolution of Opsin Genes in Caddisflies (Insecta: Trichoptera)
Source: Genome Biol Evol. 2024 Aug 23;16(9):evae185. doi: 10.1093/gbe/evae185 (PMC11381090; doi:10.1093/gbe/evae185)

CDS Tree

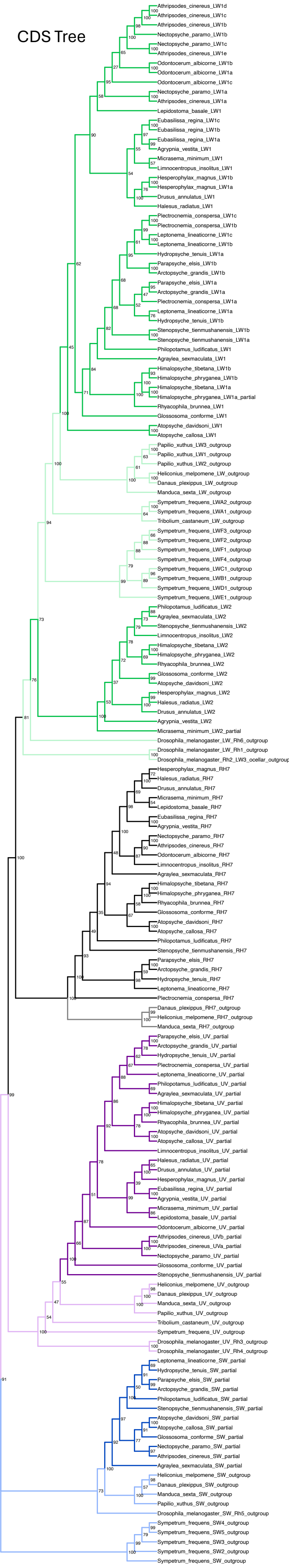

Peptide Tree

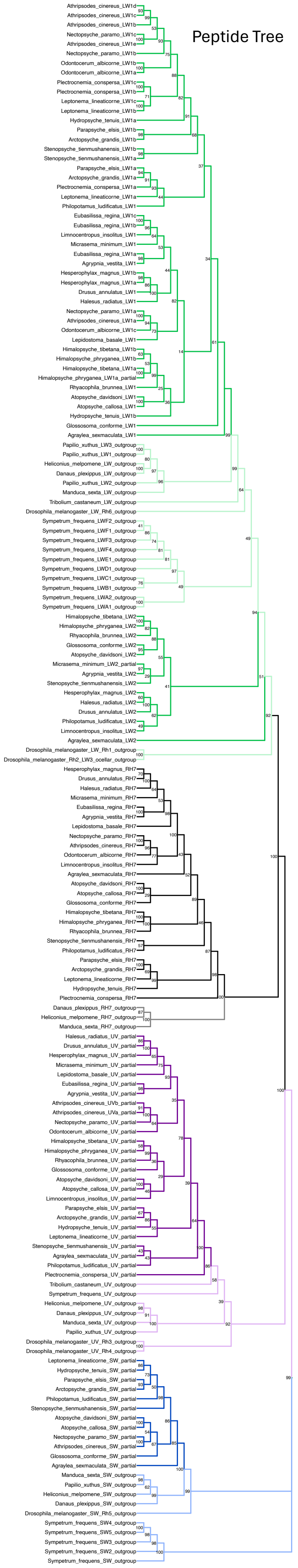

Supplement: evae185_Supplementary_Data [file evae185_supplementary_data.zip › FigureS2.pdf]
